# Identification of Connexin 26 on Extracellular Vesicles from Human Cardiomyocytes and Plasma: Novel Insights into miRNA Loading and Oxidative Injury

**DOI:** 10.3390/ijms262010128

**Published:** 2025-10-17

**Authors:** Letizia Mattii, Alessandra Falleni, Enza Polizzi, Antonella Cecchettini, Antonietta R. Sabbatini, Manuela Cabiati, Silvia Del Ry, Valentina Casieri, Vincenzo Lionetti, Federico Vozzi, Stefania Moscato, Rosalinda Madonna

**Affiliations:** 1 Histology Division, Department of Clinical and Experimental Medicine, University of Pisa, 56126 Pisa, Italy; enza.polizzi@unipi.it (E.P.); stefania.moscato@unipi.it (S.M.); 2 Biology Division, Department of Clinical and Experimental Medicine, University of Pisa, 56126 Pisa, Italy; alessandra.falleni@unipi.it (A.F.); antonella.cecchettini@unipi.it (A.C.); 3 Institute of Clinical Physiology, National Research Council, 56124 Pisa, Italy; manuela.cabiati@cnr.it (M.C.); silvia.delry@cnr.it (S.D.R.); federico.vozzi@cnr.it (F.V.); 4 Laboratory of Biochemistry, Department of Pathology, University of Pisa, 56126 Pisa, Italy; antonietta.sabbatini@unipi.it; 5 Laboratory of Basic and Applied Medical Sciences, Unit of Translational Critical Care Medicine, Interdisciplinary Research Center “Health Science”, Scuola Superiore Sant’Anna, 56127 Pisa, Italy; valentina.casieri@santannapisa.it (V.C.); vincenzo.lionetti@santannapisa.it (V.L.); 6 UOSVD Anesthesia and Intensive Care, Fondazione Toscana “G. Monasterio”, 56124 Pisa, Italy; 7 Cardiology Division, Department of Pathology, University of Pisa, 56124 Pisa, Italy; rosalinda.madonna@unipi.it

**Keywords:** connexin 26 (Cx26), extracellular vesicles, miRNA, cardiomyocytes, oxidative stress, intercellular communication

## Abstract

Connexin 26 (Cx26), a gap junction protein, is poorly understood in the context of cardiac milieu, including extracellular vesicles (EVs). Here, we report for the first time the presence of Cx26 on EVs obtained from human induced pluripotent stem cell-derived cardiomyocytes and human plasma. Using an in vitro model of oxidative stress and apoptosis in dH9c2 cardiomyocytes, we observed a significant decrease in Cx26 levels in EVs released by injured cells, accompanied by changes in EV concentration, particularly in exosomes. Our findings revealed that Cx26 modulates the selective loading of specific microRNAs, namely miR-1 and miR-30a, into EVs, suggesting a novel non-canonical, gap junction-independent role of Cx26 in EV-mediated cardiac signaling. Analysis of plasma EVs from healthy donors confirmed the presence of Cx26-positive EVs of cardiomyocyte origin, indicated by co-staining with cardiac troponin T. These findings suggest that further studies on the measurement of Cx26 on circulating EVs from patients with ischemic heart disease and heart failure are warranted to clarify its potential as a biomarker for cardiomyocyte injury in cardiomyopathies with oxidative stress and apoptosis.

## 1. Introduction

Intercellular communication is essential for tissue coordination and homeostasis, particularly in complex multicellular systems like the heart. In addition to direct cell–cell contact through gap junctions, emerging evidence highlights the role of long-range signaling mechanisms, including communication mediated by extracellular vesicles (EVs). EVs are vesicles ranging in size from 30 nm to 4 μm that carry molecular cargo, including proteins, lipids, and microRNAs (miRNAs), to recipient cells, often at distant sites [1]. This form of communication is now recognized as a key regulator of physiological processes and pathophysiological responses within the cardiovascular system, including cardiac development, injury, and repair [2,3]. In this context, connexins (Cxs), traditionally known to form gap junction channels, have more recently been implicated in the EV function as well. Gap junctions are plaques containing hundreds to thousands of intercellular channels. Each channel is formed by the oligomerization of six Cxs at the cell surface creating a connexon (hemichannel) that docks with a connexon on a neighboring cell. This structure establishes direct intercellular communication, allowing the exchange of 1–1.8 kDa molecules, such as electrical signals, small metabolites, and second messengers. Gap junctions among heart cells, including endothelial cells, stromal cells, and cardiomyocytes, are essential for cardiac function by allowing the exchange of chemical signals. In particular, gap junctions between cardiomyocytes are predominantly localized at intercalated discs where they mediate electrical cell–cell coupling and thus support the propagation of electrical signals throughout the heart. Among the 21 known human Cxs, Cx26 and Cx43 are expressed throughout the myocardium, whereas Cx40, Cx45 and Cx46 are selectively expressed in specific regions of the heart. Cx43, extensively investigated, is mainly localized at intercalated where it forms gap junction channels between cardiomyocytes, enabling both electrical impulse propagation and signal transduction [4,5,6]. More recently, Cx43 has been implicated in cardiac development, fibrosis, and tissue repair, via formation of hemichannels on the EV surface [7,8,9]. Conversely, the role of Cx26, which has only recently been identified in cardiomyocytes [10], remains largely unexplored. Cx26 is absent from intercalated discs and does not appear to contribute to canonical gap junction formation in cardiomyocytes; instead, its subcellular distribution suggests a potential non-canonical gap junction-independent function [11]. Notably, Cx26 has been identified within intracellular compartments such as multivesicular bodies (MVBs), precursors of EVs, and on the surface of EVs released from rat cardiomyocytes. These observations suggest that cardiac Cx26 may participate in EV-mediated intercellular signaling rather than direct cell–cell coupling.

To test this hypothesis, we first investigated the presence of Cx26 in EVs derived from human plasma and cardiomyocytes from human induced pluripotent stem cells (hiPSCs). Then, using an in vitro model of cardiac injury involving oxidative stress and apoptosis, we assessed the modulation of vesicular Cx26 and its potential role in influencing EV cargo composition, with a particular focus on miRNA loading. This was motivated by the presence of multiple predicted RNA-binding motifs within the Cx26 protein sequence [12] and previous evidence of a similar role for Cx43 [9]. We have for the first time revealed a novel non-canonical gap junction-independent role of Cx26 in EV-mediated cardiac signaling that could be involved in intercellular communications underlying oxidative stress cardiac injury. Thus, the measurement of circulating vesicular Cx26 in IHD patients may help clarify its potential as a biomarker for cardiomyocyte injury in cardiomyopathies with oxidative stress and apoptosis, including ischemic heart disease (IHD) and heart failure (HF). For this reason, although preliminary, the results of this study are significant and provide strong motivation for further clinical investigation.

## 2. Results

### 2.1. Human Cardiomyocytes, as well as Cardiac Extracellular Vesicles from hiPSC-CMs and Human Plasma Display Cx26 Under Normal Conditions

**Figure 1:**
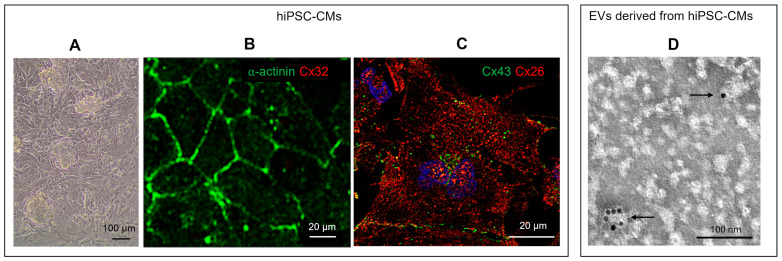
Characterization of hiPSC-CMs and Cx26 expression in hiPSC-CM derived EVs. (**A**) hiPSC-CMs at light inverted microscopy. (**B**,**C**) Representative confocal images of cardiomyocytes derived from hiPSCs cultured in chamber slides and subjected to double immunofluorescence: (**B**) green immunopositive reaction for sarcomeric α-actinin and no visible red immunonegative reaction for Cx32, (**C**) green immunopositive reaction for Cx43 and red immunopositive reaction for Cx26; nucleus (DAPI, blue). Image in (**C**) was acquired in one Z-plane (8th of 30). (**D**) Representative TEM image of Cx26 immunogold analysis on EVs from hiPSC-CMs, performed with 10 nm gold particles conjugated to anti-rabbit secondary antibody. Arrows point to Cx26 immunopositive EVs.

Cultured hiPSC-CMs (Figure 1A) were characterized for their cardiac phenotype by the presence of spontaneous beating and the immunopositivity for specific cardiomyocytic markers as sarcomeric α-actinin and Cx43 (Figure 1B,C). Cx32, a Cx expressed by liver and salivary glands but not by the heart [10], was used as a negative control and was not detected by immunofluorescence (Figure 1B). Cx26 was expressed by hiPSC-CMs at both the cytoplasm and plasma membrane levels, as demonstrated by the positive red fluorescent signal (Figure 1C). TEM-immunogold analysis further revealed the expression of Cx26 on the surface of some EVs isolated from the culture supernatant of hiPSC-CMs (Figure 1D). In particular, Cx26 was observed on EVs smaller than 100 nm in size.

**Figure 2:**
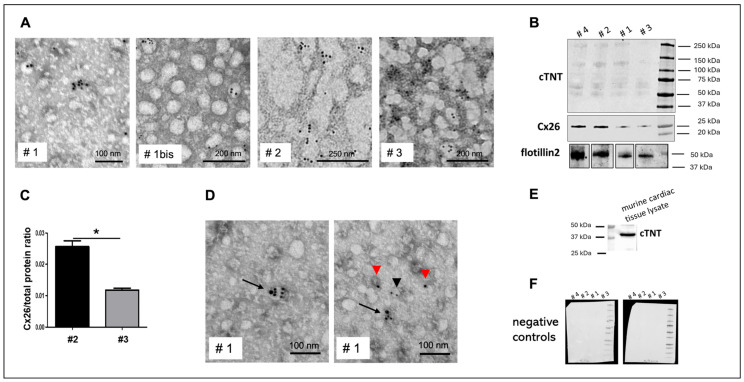
Cx26 expression in human cardiac-derived plasma EVs. (**A**) Representative TEM images with 10 nm immunogold for Cx26 on EVs from frozen human plasma of donors 1–3 (plus unfrozen donor 1 sample, 1 bis). (**B**) Representative Western blot of EVs from human plasma of donors 1–4 showing Cx26 with flotillin 2 as EV marker (n = 2) and cTNT as cardiomyocyte marker (n = 1). (**C**) Semi-quantitative analysis of Cx26 levels in EVs from donors 2 and 3 (n = 2), * *p* < 0.05. (**D**) Representative double immunogold TEM images (10 nm gold for Cx26, 20 nm gold for cTNT) on EVs from donor 1; arrows indicate EVs positive for both Cx26 and cTNT; black arrowhead points to positive EVs only for Cx26 while the red ones only for cTNT. (**E**) cTnT blot of murine cardiac lysate. (**F**) Western blotting of EVs from human plasma of donors 1–4 probed with secondary antibodies only, anti-rabbit (left) or anti-mouse (right).

EVs isolated from the plasma of four donors were analyzed for Cx26 expression using transmission electron microscope (TEM) immunogold labeling, Western blot, or both (Figure 2). Cx26 could be detected on plasma EVs using either immunogold labeling or Western blot. TEM-immunogold labeling enabled the precise visualization and confirmation of Cx26 localization on EVs with a diameter smaller than 100 nm, thereby substantiating their classification as exosomes. Moreover, as shown in the representative TEM images of Figure 2A, EVs extracted from fresh, unfrozen plasma (identified as ‘bis’) appeared to maintain their morphology and individuality better than those derived from frozen plasma, which appeared less well-defined and more prone to aggregation. Nevertheless, pre-freezing the plasma did not alter Cx26 expression, as demonstrated by immunoblotting analysis, which showed the band reactivity at 26 kDa. Flotillin 2, used as an exosome-specific marker [13], was present in all samples (Figure 2B). Given that our primary interest was qualitative, specifically, the presence or absence of Cx26 on plasma-derived EVs, an equal volume of EV eluate was loaded for each sample in the Western blot analysis. However, due to notable differences in band intensity at ~26 kDa between samples, a semi-quantitative assessment of Cx26 expression was subsequently performed on two out of the four samples, normalized to the total protein content as visualized by the stain-free blot. The different intensities of the 26 kDa bands displayed in the blotting by the two samples were confirmed by the semi-quantitative analysis (Figure 2C).

To identify the presence of plasma EVs derived specifically from cardiomyocytes rather than from other tissues, we investigated the presence of cardiac troponin T (cTNT) as a cardiomyocyte-specific marker. Immunoblotting and immunogold labeling confirmed the presence of cTNT in plasma-derived EVs (Figure 2B,D). In particular, TEM analysis (Figure 2D) revealed double immunostaining for Cx26 and cTNT in various EVs, demonstrating that a large subset of Cx26-positive plasma EVs originated from cardiomyocytes.

Interestingly, in the immunoblotting of plasma EV lysates, the mouse anti-cTNT antibody reacted differently than in immunoblotting of murine cardiac tissue lysates, which showed the expected band at 39 kDa (Figure 2E). Specifically, in EV immunoblotting, the anti-cTNT antibody displayed multiple bands with a molecular weight slightly above 50 kDa (Figure 2B). These bands were specific and not due to secondary antibody nonspecificity, as demonstrated by immunoblotting performed using only the secondary antibodies, which yielded no signal (Figure 2F).

### 2.2. Oxidative Stress Increases the Production of EV-Derived from Rat Cardiomyocytes

**Figure 3:**
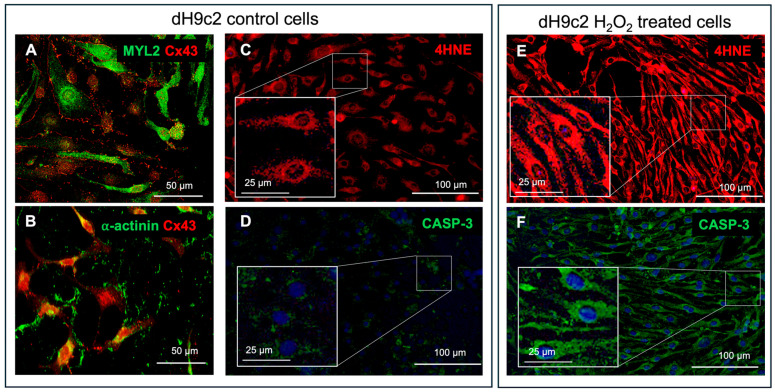
Characterization of dH9c2 cardiomyocytes under normal and oxidative stress conditions. Representative images of double immunofluorescence for cardiac muscle isoform of MYL2 ((**A**), green), sarcomeric α-actinin ((**B**), green) and Cx43 ((**A**,**B**), red). (**C**–**F**) Cell injury evaluation induced by H_2_O_2_ treatment. Representative images of immunofluorescence for 4HNE in control ((**C**), red) and H_2_O_2_ treated cells ((**E**), red), and CASP-3 in control ((**D**), green) and H_2_O_2_ treated cells ((**F**), green). In (**D**,**F**) the nuclei are marked in blue with DAPI. The brightness and contrast of the magnified images in the squares were increased in the same way for all enlargements, in order to facilitate the identification of the immunofluorescence reaction.

Having demonstrated Cx26 expression on EVs from human cardiomyocytes and plasma, we next used differentiated rat H9c2 cardiomyocytes (dH9c2) to assess whether cellular injury modulates EV production and Cx26 levels on EVs. Cardiac phenotype was confirmed by immunostaining for α-actinin, MYL2, and Cx43 (Figure 3A,B). In this established system, where EVs are known to express Cx26 [11], oxidative stress and apoptosis were triggered via H_2_O_2_ exposure (see Section 4.1), confirmed by 4-hydroxy-2-nonenal (4HNE) and caspase 3 (CASP-3) immunoreactivity (Figure 3C–F).

**Figure 4:**
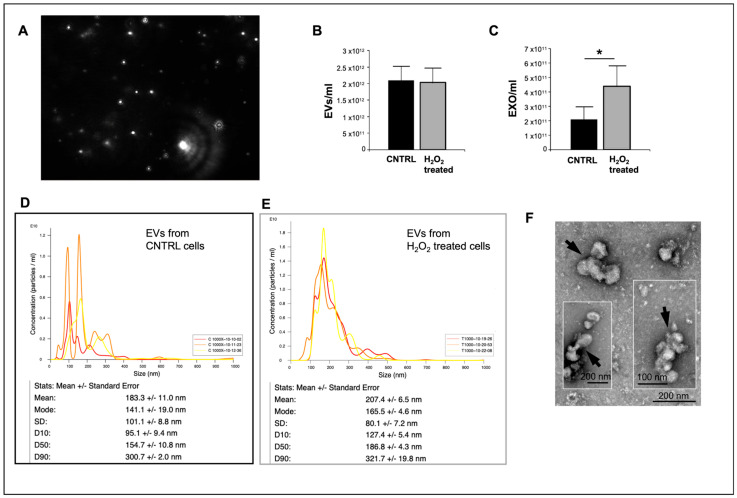
Characterization and sizing of EVs derived from dH9c2 cardiomyocytes. (**A**) a screenshot of video from NanoSight LM10 showing EVs. (**B**,**C**) Concentration of EVs derived from control or H_2_O_2_ treated cells: (**B**) total EVs, n = 3 or (**C**) exosomes, n = 3, * *p* < 0.05. (**D**,**E**) Characterization for the size and the number of EVs derived from control (**D**) and H_2_O_2_-treated (**E**) cells. (**F**) Representative TEM images of EVs from treated cells. Arrows point to aggregates of four or more EVs.

The analysis of EV concentration using the NanoSight LM10 technology (Figure 4A) demonstrated that the number of total EVs in the supernatant from control cells or cells under oxidative stress did not change (Figure 4B). However, we also focused on exosomes, the EVs with a smaller diameter. Considering the exosomes, it was observed that their concentration increased when they originated from cells under oxidative stress compared to those from control cells (*p* = 0.014, n = 3, Figure 4C). Moreover, as shown in Figure 4D,E, NanoSight analysis of size distribution revealed that the mean size of EVs derived from H_2_O_2_-treated cells was larger than that of EVs from control cells (207 ± 6.5 nm vs. 183.3 ± 11 nm). Additionally, only 10% of EVs (OD10) had a size smaller than 127 nm or 95 nm. These results contrast with our previous findings, obtained with TEM analysis of EVs from dH9c2 control cells [11], which showed that the majority of EVs had a size of 30 to 50 nm. This apparent contradiction could be explained by assuming that in our present samples, EVs tended to form aggregates of five or six elements, which were instead recognized by the NanoSight as single vesicles. This hypothesis was supported by TEM analysis of the same samples, albeit collected in a separate experiment, which showed a certain degree of EV aggregation (Figure 4F) and by the absence of DNase pretreatment, a step employed to eliminate extracellular DNA that can induce aggregation of exosomes [14]. Nevertheless, although some uncertainty may persist regarding the precise quantification of EVs, potentially underestimated in this case by NanoSight analysis, there is greater confidence that the number of exosomes from the treated samples increased compared to those from controls.

### 2.3. Oxidative Stress Modulates the Expression of Cx26 and miRNA Cargo in Rat Cardiomyocytes-Derived Extracellular Vesicles

We hypothesized that Cx26 might constitute connexons/hemichannels on EVs and could be involved in the modulation of the EV cargo, namely in terms of miRNAs, due to the presence of several predicted RNA-binding motifs in the Cx26 sequence [14] and to the previous study of Martins-Marques on Cx43 [9]. To address this issue, we first selected the following miRNAs, miR-1, miR-208a and miR-30a, as they were previously found to be expressed in H9c2 cells and in their EVs [15,16,17]. As we used H9c2 cells differentiated into cardiomyocytes instead of undifferentiated cells, we had to confirm the presence of these miRNAs in EVs derived from the differentiated cells. We then evaluated whether the expression of Cx26, as well as the miRNA cargo, changed in EVs derived from dH9c2 cells under oxidative stress. Oxidative stress plays a key role in several cardiovascular diseases, also altering the production of many miRNAs, including miR-1 [18,19].

**Figure 5:**
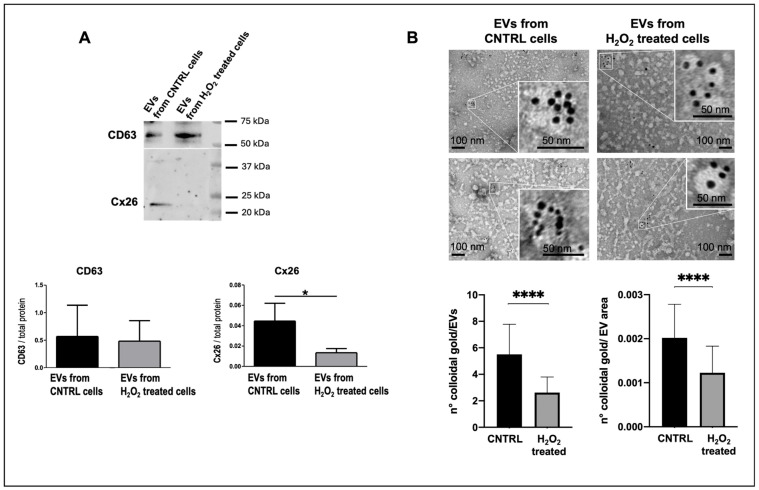
Cx26 expression in EVs derived from dH9c2 cardiomyocytes under normal and oxidative stress conditions. (**A**) Western blotting for Cx26 and CD63 of EVs from not treated (CNTRL) or H_2_O_2_-treated, under oxidative stress, cells. The upper panel shows a representative immunoblotting of EV lysates, while the lower panel shows the semi-quantitative analysis from six independent blotting experiments. * *p* < 0.05 vs. control. (**B**) TEM-Immunogold analysis of EVs from CNTRL and H_2_O_2_-treated cells. The upper panel shows representative images of EVs immunopositive for Cx26, where the aggregation state of the EVs, which frequently occurs, is particularly evident in the images of the control group. The lower panel presents the relative semi-quantitative analysis expressed as the number of colloidal gold particles for EV or for EV area. **** *p* < 0.0001 vs. control.

Western blotting analysis of EV lysates demonstrated for the first time that Cx26 decreased in EVs derived from injured cardiomyocytes. Specifically, the amount of Cx26 in EVs from injured cells was reduced more than threefold compared to EVs from control cells (*p* = 0.04, n = 6, Figure 5A). The ratio of CD63 expression to total protein content remained unchanged in EVs from both control and treated cells. TEM images confirmed the immunoblotting results and further showed that (1) the number of EV presenting Cx26 was lower when EVs derived from cells under oxidative stress than when derived from control cells and (2) each observed EVs had fewer gold particles than those derived from control cells, as demonstrated by the number of colloidal gold/EV area and by the number of colloidal gold /EVs, respectively (Figure 5B). According to the results obtained in this study with EVs from hiPSC-CMs (Figure 1D) and in a previous study with EVs from dH9c2 [11], the EVs with higher Cx26 content are those with a diameter typical of exosomes (Figure 5B).

The results obtained from profiling the expression levels of three miRNAs in EV lysates derived from control or H_2_O_2_-treated dH9c2 cells indicated for the first time that miR-1, miR-208a, and miR-30a were expressed by H9c2 cells even after differentiation into cardiomyocytes, and that they are present in the EVs released by these cells. Moreover, the results showed a trend toward changes in the expression of miR-1 and miR-280a that can be observed in the EVs from cells under oxidative stress compared to those from control cells. In particular, miR-1 decreased, while miR-280a increased in EVs from cells under oxidative stress (mean ± SEM: 0.855 ± 0.305, for miR-1; 2.812 ± 2.704, for miR-280a; 1.929 ± 1.342, for miR-30a) compared to EVs from control cells (2.482 ± 1.276, for miR-1; 0.794 ± 0.317, for miR-280a; 1.480 ± 0.722, for miR-30a), although in a not significant manner.

### 2.4. Cx26 Modulates the Selective Sorting of miR-1 and miR-30a into EVs from H9c2 Cells

**Figure 6:**
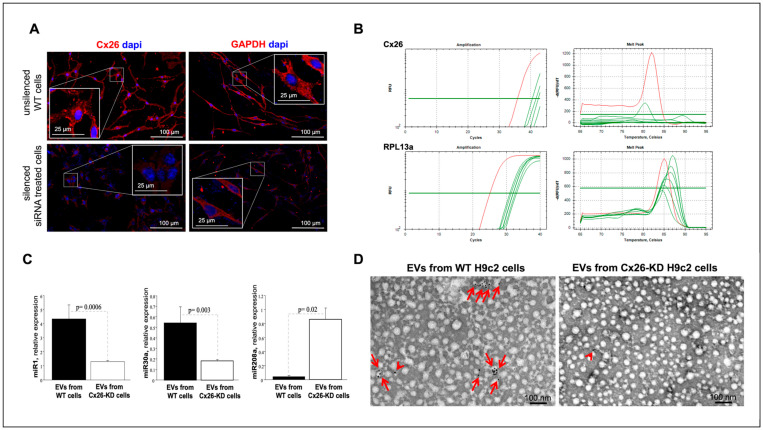
Unsilenced WT and Cx26-silenced H9c2 cells release EVs with different miRNAs cargo. (**A**) Representative images showing Cx26 and GAPDH red immunopositive staining in scrambled siRNA-silenced cells (WT, top), and immunonegative staining in cells treated with related siRNA (KD, bottom). (**B**) RT-PCR analysis. Real-time PCR results for Cx26 mRNA (threshold cycles, Ct values, and corresponding melting peaks [upper panels]) and RPL13a mRNA (threshold cycles, Ct values, and corresponding melting peaks [lower panels]) in WT (red) and Cx26-KD (green) cells. (**C**) Relative expression levels of miR-1, miR-30a, and miR-280a carried by EVs released by WT cells (n = 3, black bar) or Cx26-KD cells (n = 8, white bar). (**D**) Representative micrographs of immunogold for Cx26 in EVs from WT and Cx26-KD cells. Red arrows point to gold particles on single or grouped EVs from WT cells. Arrowhead points to unspecific gold particles non-bound to EVs from Cx26-KD cells.

To examine whether Cx26 is mechanistically involved in the EV-miRNA expression under normal conditions, we used RNA interference (siRNA) technology to knock down (KD) gene expression of Cx26 in H9c2 cardiomyoblasts not fully differentiated into cardiomyocytes, as detailed in Materials and Methods. Cx26 siRNA did not affect cell morphology or cell proliferation (Figure 6A) but triggered a substantial (90%) reduction in Cx26 protein and mRNA expression in KD-H9c2 cells (Figure 6A,B) compared to control cells. By contrast, serving as a sequence-unrelated control, scrambled siRNA did not exert any detectable effect (Figure 6A). The reliability of the result was ensured by the silencing of GAPDH mRNA achieved in the same experiments as well (Figure 6A). We analyzed the abundance of miR-1, miR-208a and miR-30a on EVs derived from both Cx26-KD H9c2 cells and Cx26-expressing H9c2 scrambled cells. miR208a expression in EVs from H9c2 was substantially increased after transfection with siRNA against Cx26 compared to scrambled siRNA, while the expressions of miR1 and miR30a were substantially suppressed (Figure 6C). Importantly, EVs from Cx26-KD cells contained a very low amount of Cx26 (Figure 6D), as expected from the successful silencing of Cx26 in the H9c2 cells (Figure 6A,B) that released these EVs.

## 3. Discussion

Our study provides novel insights into the role of Cx26 in EV-mediated cardiac signaling involved in intercellular communication during cardiac injury caused by oxidative stress.

Cx26 on EV membranes could form hemichannels, influencing cargo loading or facilitating the transfer of various cargo molecules (e.g., ions, nucleotides, amino acids, sugars, mRNA, and miRNA) into target cells. Since at the cardiac level Cx26 is absent from intercalated discs, this EV-associated role may represent its primary mode of action in cardiomyocytes, distinct from the gap junction-dependent functions typically attributed to the other cardiac Cxs predominantly localized at intercalated discs. Furthermore, circulating vesicular Cx26 may serve as a biomarker of oxidative stress cardiac injury.

In this study, we propose that Cx26-positive plasma EVs predominantly originate from cardiomyocytes, since in other Cx26-expressing tissues, such as gut smooth muscle [20] or cochlear epithelium [21], Cx26 functions primarily in gap junctions rather than in EVs. To further confirm a cardiac origin, we performed double immunogold labeling for Cx26 and cTNT, a well-established marker of heart-derived EVs [10,22], demonstrating co-localization on circulating EVs.

Notably, we detected cTNT on the EV surface, as immunogold labeling was performed on intact, non-permeabilized vesicles, indicating that the anti-cTNT antibody recognizes an epitope accessible on the EV exterior. This is noteworthy because it implies that this antibody could be used to isolate cardiomyocyte-derived EVs from plasma, addressing the current lack of truly cardiomyocyte-specific EV surface markers. Recent studies have proposed CD172a and Ldb3 as candidate cardiomyocyte EV markers [22,23]. However, CD172a is also expressed by certain leukocyte subsets, requiring complex multicolor flow cytometry with a back-gating strategy to distinguish cardiac EVs from contaminants. Ldb3 is enriched in cardiomyocyte-derived EVs, but its precise localization on or within EVs remains to be validated before it can be reliably used for isolating cardiomyocyte-derived EVs. Immunoblotting analyses revealed multiple immunoreactive bands at molecular weights exceeding that of the expected free cTNT. This suggests that cTNT in EVs exists not only as a free protein but may also be complexed with other molecular assemblies. This finding aligns with a recent report that cardiomyocytes release troponin in association with sarcomeric proteins via autophagy-derived “exospheres” (large extracellular vesicles) as part of cellular homeostasis [24]. In the same report, it is suggested that the incorporation of cTNT into molecular complexes within EVs may reflect specific cargo packaging or sorting mechanisms, influenced by the physiological or pathological status of the parent cells.

Alterations in Cx26 levels on cardiomyocyte-derived EVs could reflect cardiomyocyte injury or dysfunction as previously proposed for Cx43. Specifically, previous work demonstrated that myocardial infarction alters Cx43 content in cardiomyocyte-derived EVs [8]. To investigate the effect of oxidative stress and apoptosis on Cx26 abundance in cardiomyocyte-derived EVs, we employed an in vitro model using dH9c2 cells treated with H_2_O_2_. Our data showed that injured cells released EVs with significantly reduced levels of Cx26 compared to controls, suggesting the involvement of EV-associated Cx26 in cardiomyocyte injury. Our analysis of plasma EVs from healthy human donors was primarily qualitative, aiming to assess the presence of Cx26 on plasma EVs. However, Western blot results revealed that the four examined samples exhibited variability in EV-Cx26 levels despite donors having no known acute or chronic diseases, including cardiac dysfunction. This variability may be attributed to the sensitivity of plasma EV-Cx26 levels to subclinical or preclinical cellular alterations, as well as to individual characteristics such as age, sex, weight, and lifestyle factors. Therefore, it will be crucial to study larger cohorts, including patients with established cardiac pathologies, to evaluate whether plasma EV-Cx26 variations may have prognostic value on disease progression. Martins-Marques et al., based on their previous findings that Cx43 facilitates EV communication with recipient cells [7] and mediates miRNA sorting in EVs derived from human embryonic kidney or cervix epithelial cells [9], proposed that decreased Cx43 levels on plasma EVs could impair intercellular transfer of metabolites, signaling molecules, and miRNAs, consequently modulating secreted miRNA profiles and potentially acting as a cardioprotective mechanism. Our findings raise the possibility that analogous mechanisms involving Cx26 may exist, which warrants further investigation. Our data in Cx26 silenced H9c2 cardiomyoblasts clearly demonstrate that Cx26 is involved in miRNA recruitment. Indeed, among the three miRNAs analyzed, levels of miR-1 and miR-30a were significantly reduced in EVs derived from cells silenced for Cx26, whereas levels of miR-280a were increased. Although the cells utilized for silencing experiments are not fully differentiated cardiomyocytes, as specified in the Materials and Methods, they remain widely used as a cardiomyocyte cellular model [25,26,27]. We anticipate that Cx26’s role in miRNA loading is retained upon differentiation. EV connexin-mediated miRNA incorporation has been well documented for Cx43 in multiple cell types [9], supporting the likelihood that Cx26 similarly contributes to selective miRNA recruitment into EVs in mature cardiomyocytes. Contrary to what we expected, considering the role of Cx26 on EVs, miRNA expression analysis after oxidative stress injury showed only a trend toward decreased miR-1 in EVs from cells under oxidative stress. The absence of statistical significance may be due to the limited number of experimental replicates or to the specific experimental conditions employed, such as H_2_O_2_ incubation time or concentration, which, although sufficient to induce oxidative stress and apoptosis, may not have been adequate to produce a significant alteration in miRNA cargo.

Interestingly, despite the reduced Cx26 levels on EVs from damaged cells without changes in cellular Cx26 protein levels, cellular injury induced increased production of EVs, specifically exosomes. The increased production of EVs following cellular injury is consistent with previous studies reporting elevated EV shedding under inflammation and hypoxia [28,29]. These data suggest that Cx26’s role on EVs is specific and operates independently of both the overall number of EVs produced and the amount of Cx26 present in the donor cells.

Our findings expand the current understanding of connexin biology, particularly for Cx26, whose cardiac function has remained largely unexplored. In addition, our work highlights important methodological advances. We have observed that for accurate EV characterization by TEM, the use of pre-frozen plasma or cell culture supernatant should be avoided. We adopted a differentiated EV isolation protocol, tailoring the method to the specific sample type and downstream analyses. This approach ensured the optimal analysis of EVs and their cargo composition for each experimental application, including immunoblotting, TEM immunogold, and RT-PCR. Such methodological rigor is essential to minimize variability and potential artifacts, thereby strengthening the reliability of EV analyses.

This “proof-of-concept” study offers novel perspectives on the function of Cx26 in the heart, highlighting its significant role in EV-mediated signaling.

In summary, we have provided new insights on a novel non-canonical, gap junction-independent role of Cx26 in EV-mediated cardiac signaling in an in vitro model of oxida-tive stress and apoptosis. This “proof-of-concept” study suggests that circulating EV-asso-ciated Cx26 may be an attractive biomarker of cardiomyocyte damage in cardiomyopa-thies characterized by oxidative stress and apoptosis, such as IHD and HF. Further in vivo preclinical studies in HF animal models, together with clinical trials, are needed to explore the functional effects of Cx26-containing EVs and their relevance in patients with HF. Such research will be crucial to better elucidate their involvement in cardiac physiology and pathology.

## 4. Materials and Methods

### 4.1. Cell Cultures

Human induced pluripotent stem cells (hiPSCs, Gibco^®^ Episomal hiPSC Line, Thermo Fisher Scientific, Waltham, MA, USA) were cultured and differentiated in cardiomyocytes on Vitronectin (VTN-N, Gibco A14700)-coated flasks with Essential 8^®^ Flex basal medium (Gibco, A28583-01) + Supplement (Gibco, A28584-01) + 1% Penicillin/Streptomycin (P/S) (referred to as Complete Flex, ThermoFisher). Revita (Gibco A2644501-01) was added to Complete Flex (5 μL of Revita/mL of medium) just for the thawing and passaging of cells for 24 h. Medium was refreshed every 2 days, and at 70–80% confluence, cells were gently expanded using Versene (Gibco, 15040-033) without destroying cell clusters. The cell seeding density was 20,000 cells/cm^2^. To start the differentiation protocol, clusters of hiPSCs (70–80% of confluence) were rinsed twice with Dulbecco’s phosphate-buffered saline (DPBS) without calcium and magnesium (DPBS-/-), then detached and singularized with TrypLE (Gibco, A12177-01, diluted in Versene or DPBS-/-). Cells were seeded in VTN-N-coated flasks (seeding density of 20,000 cells/cm^2^), and the medium was replaced every 2 days until 50–60% confluence. Then, the differentiation protocol started and continued for 12 days using the PSC Cardiomyocyte Differentiation Kit (Gibco, A29212-01). Day 0: Complete Flex was removed, and Medium A (Gibco, A29209-01) + 1% P/S was added. Day 2: Medium A was removed, and Medium B (Gibco, A29210-01) + 1% P/S was added. Day 4: Medium B was replaced with Cardiomyocyte Maintenance Medium (Gibco, A29208-01) and 1% P/S (referred to as Maintenance medium). The medium was refreshed on days 6, 8, and 10 till cells started to beat. On day 12, the cells were incubated with ROCK inhibitor (ROCKi, Y-27632) (Merck, Darmstadt, Germany, SCM075) (final concentration 10 μM in Maintenance medium) for 1 h at 37 °C. Cells were then detached using TrypLE (ThermoFisher), and after centrifugation at 300× *g* for 5 min, the pellet was resuspended in Cryopreservation medium (Gibco, A26444-01) or Fetal Bovine Serum (FBS) supplemented with 10% dimethyl sulfoxide (DMSO) and stocked in liquid nitrogen. HiPSC differentiated into cardiomyocytes (hiPSC-CM) were characterized by the presence of beat, expression of specific cardiac markers as c-αactinin and Cx43, and by the absence of expression of non-cardiac specific marker such as Cx32 (Figure 1A–C).

H9c2 myoblasts, purchased from American Type Culture (ATCC, Rockville, MD, USA) and differentiated into cardiomyocytes (dH9c2), were used to build the in vitro model of cardiac injury. H9c2 cells were cultured in high glucose Dulbecco’s modified Eagle’s medium (DMEM, ATCC) supplemented with 10% heat-inactivated fetal bovine serum (FBS) and 1% penicillin-streptomicin (Merck, Darmstadt, Germany) under 95% air and 5% CO_2_ at 37 °C. Differentiation into cardiomyocytes was performed in DMEM containing 1% FBS and all trans-retinoic acid (RA; Merck) supplementation (50 nM) for 10 days, as previously described [12], and confirmed by immunofluorescence positivity for c-αactinin, MYL2 and Cx43 (Figure 3A,B). dH9c2 injured model was obtained inducing oxidative stress by H_2_O_2_ treatment as previously described for H9c2 cells [30,31] with some adjustments, namely, dH9c2 cells were treated with 300 μM of H_2_O_2_ for 24 h. This treatment, according to MTT assay, showed approximately 50% toxicity for control dH9c2 cells. Then the cell injuries induced by H_2_O_2_ treatment, such as oxidative stress and apoptosis, were verified by both the expression of 4HNE, considered a specific sensitive oxidative stress marker and the expression of CASP-3 (Figure 3C–F).

For immunofluorescence analysis, dH9c2 cells cultured in chamber slides, were fixed in 1% formaldehyde/PBS for 10 min at 4 °C and stored at 4 °C. All experiments were performed on the cells at 70–80% subconfluence.

For studying EVs, in the model of cardiac injury, a culture medium containing a FBS previously ultracentrifuged at 100,000× *g* for 2 h, to eliminate serum EVs, was used at the last medium change. Then supernatants of cell cultures were harvested, centrifuged at 200× *g* to remove cell debris and further centrifuged at 2000× *g* for 30 min at 4 °C or filtered through 0.8 µm pore size syringe filters (Millex^®^-AA, Merck) to remove apoptotic bodies. Then, the supernatants were immediately treated for EV extraction or frozen in aliquots at −80 °C, until further processing, according with previous procedures [32,33].

### 4.2. Human Samples

The use of human samples was conformed to the principles outlined in the Declaration of Helsinki and was approved by the Bioethics Committee of the University of Pisa (Rev 40/2023 approved on 29 September 2023). Five to ten ml of venous blood samples were taken from healthy volunteers without any known acute or chronic diseases (n = 4, 3 women-1 man; age = 50–67) and collected into EDTA or sodium citrate. Plasma samples, obtained by 10 min at 3000 rpm centrifugation at 4 °C, were filtered using 0.8 µm filters, to remove remaining platelets and apoptotic bodies, and immediately treated for EV extraction or frozen in aliquots at −80 °C, until further processing.

### 4.3. Immunofluorescence (IF) Staining of Cultured Cell Lines

Fixed hiPSC-CM, dH9c2 and H9c2 cells were hydrated in water, washed in PBS, and then incubated with 0.2% triton-X100/PBS for antigen retrieval. Successively, samples were incubated with blocking solution (BS: 0.1% Tween 20, 0.25% BSA in PBS) for 1 h and then, overnight at 4° C, with the following primary antibodies diluted in BS: mouse monoclonal anti-cαactinin (Sigma Aldrich, Merck, A7732, 1:100), rabbit polyclonal anti-Cx26 (Novus Biologicals, Centennial, CO, USA, NBP2-41304, 1:200), mouse monoclonal anti-Cx43 (Santa Cruz Biotechnologies, Dallas, TX, USA, SC-271837, 1:50), rabbit polyclonal anti-Cx32 (Invitrogen, Thermo Fisher Scientific, 710600, 1:50), rabbit polyclonal anti-GAPDH (SIGMA, Merck, G9545, 1:20,000), rabbit polyclonal anti-4HNE (BIOSS Antibodies, Woburn, MA, USA, bs-6313R, 1:100), mouse monoclonal anti-caspase 3 (BIOSS Antibodies, bsm-33199M, 1:100), mouse anti-MYL2 (Santa Cruz Biotechnologies, sc-517414, 1:50). After washing in BS, samples were incubated for 90 min at RT in the dark with fluorescent goat anti-mouse (Alexa Fluor^TM^ 488, Invitrogen, Thermo Fisher Scientific, A11001, 1:250) or donkey anti-rabbit (Alexa Fluor^TM^ 568, Invitrogen, Thermo Fisher Scientific, A10042, 1:250) secondary antibodies. For double immunofluorescence analyses, the samples were incubated in the mixture of two primary antibodies (anti-cαactinin and anti-Cx32 or anti-Cx43 and anti-Cx26 or anti-MYL2 and anti-Cx43 or anti-cαactinin and anti-Cx43) and then in the mixture of two secondary antibodies. Finally, samples were mounted with mounting medium (Prolong^TM^ Diamond Antifade Mountant with or without DAPI, Invitrogen, Thermo Fisher Scientific). The entire procedure was performed at room temperature unless specified. The negative controls for the specificity of the secondary antibodies were performed by omitting primary antibodies that were replaced by the BS solution. Immunofluorescent reactions were observed by a standard light microscope (BX43, Olympus, Hamburg, Germany) or by a confocal laser scanning microscope (TC SP8 Leica Microsystems, Mannheim, Germany) at 200×–400× magnification.

### 4.4. EV Isolation and Count

EVs were extracted from 10 mL cell supernatant or 1 mL plasma per sample using a membrane-based affinity binding step assay (EM, exoEasyMaxi kit, Qiagen GmbH, Hilden, Germany) and/or ultracentrifugation (UC), according to the manufacturer’s instructions and with previous procedures [32,33], depending on the analyzes to be performed on the collected EVs, as optimized and specified below. EV extraction using the EM kit was performed according to the manufacturer’s protocol. Briefly, plasma samples or cell supernatants were diluted with binding buffer (XBP) in a 1:1 ratio. The samples were then transferred into an ExoEasy spin column and centrifuged sequentially at 500× *g* for 2 min and at 4200× *g* for 1 min. After discarding the flow-through, 5–10 mL of wash buffer (XWP) was added to the column, followed by centrifugation at 4200× *g* for 5 min. Next, 400 µL of elution buffer (XE) was added to the column, which had been transferred to a fresh collection tube. After 1 min of incubation, the sample was centrifuged at 500× *g* for 5 min. The eluate was then re-applied to the column, incubated for 1 min, and centrifuged again at 4200× *g* for 5 min. The eluate containing EVs in XE buffer was frozen at −80 °C until further processing for Western blot or RT-PCR For transmission electron microscopy analysis, different procedures were applied depending on the source of the EVs. The EM procedure was essential for extracting EVs from plasma and hiPSC-CM supernatants, as it effectively removed most contaminating proteins that caused significant background signal in TEM observations. Subsequent ultracentrifugation was performed to eliminate the XE buffer, which also contributed to background noise. Specifically, for isolating EVs from plasma and hiPSC-CM supernatants, the eluate obtained through EM was diluted in PBS and ultracentrifuged at 100,000× *g* for 2 h at 4 °C to pellet the EVs. In contrast, ultracentrifugation alone was used for isolating EVs from H9c2 and dH9c2 cell supernatants. The EV pellet was then fixed in 2% paraformaldehyde (PFA) and processed for immunocytochemistry and negative staining at TEM. All the reactions were performed at room temperature (RT) unless otherwise specified.

EVs from the dH9c2 injury model were also analyzed for size and concentration using the NanoSight LM10 particle tracking analysis (NTA) device (NanoSight Ltd., Amesbury, Wiltshire, UK) in accordance with previous procedures [34]. Briefly, a 50 µL aliquot of eluate containing EVs derived from the supernatant of 75 culture flasks of control (C 1000) and H_2_O_2_-treated (T 1000) cells was diluted 1:1000 in PBS. Each sample was then subjected to three 60-s counts. The averages (of three counts) of the EVs as well as the particles with sizes in the range of 39.5 nm to 159.5 nm (the typical range for exosomes) were calculated, and control versus treated samples were compared.

### 4.5. EV Immunogold and Negative Staining for Transmission Electron Microscope (TEM)

EVs from human plasma and cell lines were processed following a modified protocol based on Soares et al. [7]. Briefly, 10 μL of EV-containing sample, previously fixed in 2% paraformaldehyde in 0.1 M PBS, was applied to 200-mesh formvar/carbon-coated nickel grids and incubated overnight at 4 °C in a humidified chamber. After washing with PBS, the grids were left to settle for 3 min at RT. To quench free aldehyde groups, the grids were incubated with 50 μL of glycine for 10 min, followed by blocking with PBS containing 0.1% BSA for 30 min. Primary antibody incubation was performed using rabbit anti-Cx26 (1:50 in PBS/0.1% BSA) alone or together with mouse anti-cTNT (Santa Cruz Biotechnology, sc-20025, 1:50) for double immunogold labeling, overnight at 4 °C in a humidified environment. After PBS washes, grids were incubated with 10 nm gold-conjugated anti-rabbit secondary antibody (Cytodiagnostics, Burlington, ON, Canada, AC-10-17-05) and/or 20 nm gold-conjugated anti-mouse secondary antibody (Abcam, Cambridge, UK, ab27242), both diluted 1:20 in PBS/0.1% BSA, for 1 h at room temperature. Following a final rinse in distilled water, grids underwent negative staining by applying 20 μL of 2% (*w*/*v*) uranyl acetate for 30 s.

The uranyl acetate solution was filtered through 0.45 μm polycarbonate filters before use. Excess stains were removed using filter paper, and grids were air-dried for 15 min before examination with a JEOL 100SX transmission electron microscope (Jeol, Tokyo, Japan) operating at 80 kV. Images were captured at magnifications ranging from 20,000× to 40,000× using an ATMxR80b Camera System (Advanced Microscopy Techniques, Wovburn, MA, USA). EV diameters were measured using ImageJ software (version 1.53v, NIH, Bethesda, MD, USA).

For experiments involving EVs from the dH9c2 injury model, Cx26-immunogold positive EVs were semi-quantitatively evaluated. The number of gold particles per EV was quantified from electron micrographs taken at magnifications between 15,000× and 25,000×. The EV area was measured in nm^2^ using ImageJ software. Additionally, 50 EVs immunolabeled for Cx26 were counted for each group.

### 4.6. EV Western Blot Analysis

Western blot analysis was performed on EVs derived from human plasma and from the dH9c2 injury model. Total proteins were isolated in an ice-cold lysis buffer (50 mM Tris, 2 mM EDTA, 100 mM NaCl 1% NP40) added of a protease inhibitor cocktail 1× (S8830, Merck). Samples were homogenized with a probe-tip ultrasonicator (SONOPLUS mini20, Bandelin, Berlin, Deutschland) (applying three 10 s pulses at 4.0 W) in an ice-bath. Lysates were centrifuged at 15,000 rpm for 20 min at 4 °C, and supernatants protein concentration was determined by the bicinchoninic acid assay (BCA) (Pierce™, Thermo Fisher Scientific) microplate method.

Due to their low concentration, proteins in EVs from human plasma and from dH9c2 supernatant were precipitated using Trichloroacetic Acid method (TCA). Briefly, samples were diluted in an equal volume of 20% TCA solution for 1 h in an ice-bath, vortexing 4 times during the incubation. Then samples were centrifuged at 15,000 rpm at 4 °C for 15 min and supernatant were discarded. Pellets were twice washed with acetone and centrifuged at 15,000 rpm at 4 °C for 15 min to remove TCA residues. After pellets suspension in Laemmli Buffer 4X containing beta-mercaptoethanol, equal volumes of samples, 6 μL for plasma-derived and 8 μL for dH9c2 supernatant-derived samples per lane, were separated on a 4–20% polyacrylamide gel (BioRad, Hercules, CA, USA) and electroblotted onto nitrocellulose membrane by the Trans Turbo Blot system (BioRad). After blocking in EveryBlot buffer (BioRad) for 5 min, the membranes were incubated overnight at 4 °C with the following primary antibodies singularly: rabbit polyclonal anti-Cx26 (Novus Biological, 1:500), mouse monoclonal anti-cTNT (Santa Cruz Biotech, 1:200), mouse monoclonal anti-CD63 (Abnova, Taipei City, Taiwan, MAB15170, 1:500), mouse monoclonal anti-flotillin 2 (Santa Cruz Biotech, SC-28320, 1:200), rabbit polyclonal anti GAPDH (Sigma-Merck, 1:10000). Anti-rabbit (BioRad, 170-6515) or anti-mouse (BioRad, 170-6516) HRP-conjugated antibodies (1:2000 in 5% dry fat milk in T-TBS, 1 h) were used as secondary antibodies. Immunoblots were visualized by means of a chemiluminescence reaction (Clarity Western ECL Substrate, BioRad) by ImageLabSoftware (version 6.0.1, BioRad) under a luminescent image analyzer (Chemidoc XSR+, Bio-Rad). Only bands below the saturation limit were analyzed. Semiquantitative evaluation of EV Western blot was performed by normalizing the protein band intensity to the relative lane total protein staining on blotted membrane obtained after TGX-stain free gel activation with Chemidoc XRS+, BioRad.

### 4.7. Extraction, Reverse Transcription and Real-Time PCR for EV miRNAs Expression

EVs derived from control and under oxidative stress dH9c2 cells were collected in XE buffer and treated for the quantification of their content in miR-1, miR-208a, miR-30a by means of Real-Time PCR as previously described [35]. Briefly, the extraction of miRNAs was conducted by using the miRNeasySerum/PlasmaKit (Qiagen srl, Milano, Italy) following manufacturer instructions; RNA integrity, purity, and concentration were assessed by measuring absorbance at 260, and 280 nm (NanoDrop Thermo Fisher Scientific) and applying the Beer–Lambert law (expected values between 1.8 and 2.1, for protein contamination). The EVs miRNA reverse transcription was carried out using the miScript II RT kit (Qiagen srl), starting from a 0.5/1 μg/sample in 20 μL of final reaction volume (60 min at 37 °C, 5min at 95 °C, and 4 °C for ∞). cDNA samples were diluted (1:5) and stored, as appropriate, at +4° C. Real-time PCR reactions were carried out in duplicate using a Bio-Rad C1000™ thermal cycler system (CFX-96 Real-Time detection system, Bio-Rad). To monitor cDNA amplification, a fluorogenic DNA binding dye, EvaGreen (SsoFAST EvaGreen Supermix, Bio-Rad Laboratories Inc., Hercules, CA, USA), was used. The miR-1, miR-208a, and miR-30a primer sequences were synthesized by Sigma-Merck (Milan, Italy). In particular, the GenBank accession number and the forward primer sequence (5-3) were reported in Table 1. To normalize miRNA data a common reference gene, snRNA U6 was used. All experiments were carried out according to the MIQE (Minimum Information for publication of Quantitative Real-Time PCR Experiments) guidelines [36].

### 4.8. Cx26 Knockdown in H9c2 Cells

Due to poor cell viability following Cx26 silencing in differentiated H9c2 (dH9c2) cells, we were unable to optimize the KD protocol in this cell type. Therefore, all silencing experiments were conducted in undifferentiated H9c2 cells, where the protocol was successfully optimized according to the manufacturer’s instructions. In brief, H9c2 cells (2.5 × 10^3^ cells/well) were seeded in bovine gelatin precoated (Fluka-Merck, 1% in dH_2_O) chamber slides with their culture medium and cultured at 37 °C in a humidified atmosphere containing 5% CO_2_ until reaching approximately 30% confluency. After 24 h, the culture medium was replaced with antibiotic-free medium supplemented with 10% exosome-depleted fetal bovine serum (FBS; A2720803, Gibco, Thermo Fisher Scientific). Cells were then transfected with 50 nM Cx26-specific siRNA targeting Gjb2 (Gene ID: 394266, Rattus norvegicus; Ambion, Thermo Fisher Scientific) using Lipofectamine™ RNAiMAX Transfection Reagent (Thermo Fisher Scientific, 13778030) at a final concentration of 0.25% (0.6 µL in 240 µL of transfection medium), according to the manufacturer’s instructions. Control groups included cells transfected with siRNA targeting GAPDH (4390849, Thermo Fisher Scientific) and a scrambled siRNA negative control (Negative Control No. 1 siRNA, 4390843, Thermo Fisher Scientific). KD efficiency was assessed by quantifying Cx26 mRNA levels via RT-qPCR and evaluating Cx26 and GAPDH protein expression by IF. Blinded microscopic assessment of cell density and morfphology revealed no discernible differences between wild-type and silenced cells.

H9c2 cells silenced for Cx26 were used for EV experiments. Specifically, 48 h post-transfection, the culture medium was collected for EV isolation. EVs from both Cx26-KD and control H9c2 cells were analyzed for the presence of miRNAs and Cx26 protein.

### 4.9. Extraction, Reverse Transcription and Real-Time PCR in H9c2 Cells

Wild-type (WT) and Cx26-knockdown (KD) H9c2 cells were extracted using the RNeasy Plus Micro Kit (Qiagen srl), which is specifically designed to purify RNA from small cell quantities (<5 × 10^5^). In brief, after re-suspensions, the cells were lysed in a highly denaturing guanidine-isothiocyanate–containing buffer, which immediately rapidly inactivates RNases to preserve RNA integrity ensure isolation of intact RNA. The lysates were then passed through a gDNA Eliminator spin column to remove genomic DNA. Ethanol was added to the flow-through to provide appropriate binding conditions for RNA, and then the samples were applied to a silica-based membrane (RNeasy MinElute spin column) and speeded on microcentrifuge at 12,000 RPM for 30 s; specific buffers were used to bind the RNA to the silica membrane, and contaminants were thoroughly washed away. High-quality RNA was then eluted in RNAse-free water. The total RNA sample concentration, reverse transcription and Real-time PCR reactions were performed as reported in the previous paragraph. The primer sequences of CX26 and RPL13a (used as positive control) by Sigma-Merck (Milan, Italy) were reported in Table 1.

### 4.10. Statistical Analysis

For miRNA expression the Relative quantification was performed by the Ct method using Bio-Rad’s CFX96 manager software v.3.1 (CFX-96 Real-Time PCR detection systems, Bio-Rad Laboratories Inc., Hercules, CA, USA). Statistical analysis was performed using Statview 5.0.1 Software for Windows (SAS Institute, Inc., Cary, NC, USA). Skewed variables were log-transformed before statistical analysis. Differences between more than two independent groups were analyzed by Fisher’s test after ANOVA, and relations between variables were assessed by linear regression analysis. The results were expressed as mean ± S.E.M., and a *p*-value < 0.05 was considered significant.

For additional statistical analyses, GraphPad Prism 5 software was used. A Mann–Whitney U test was performed to compare data between groups (including Cx26 expression on EVs from human plasma samples and Cx26 or CD63 expression in dH9c2 control versus H_2_O_2_-treated cells). Results were expressed as mean ± S.E.M., and *p*-values < 0.05 were considered statistically significant.

## Figures and Tables

**Table 1:** Primer sequence details of the analyzed miRNAs and genes.

Gene	Forward Primer Sequences (5′---3′)	GenBank, Accession Number	Temperature, °C
*rno-miR-1-3p* (*Mir1*)	F: TGGAATGTAAAGAAGTGTGTAT R: Universal primer	NR_032116.1	55
*rno-miR-30a-5p* (*Mir30a*)	F: TGTAAACATCCTCGACTGGAAG R: Universal primer	NR_031843.1	55
*rno-miR-208a-3p* (*Mir208a*)	F: ATAAGACGAGCAAAAAGC R: Universal primer	NR_031922.1	55
*Cx26*	F: GCTCACTGTCCTCTTCATC R: AATCGGCTTGCTCATCTC	NM_001004099	60
*RPL13a*	F: GGATCCCTCCACCCTATGACA R: CTGGTACTTCCACCCGACCTC	NM_173340.2	60

*rno-miR-1-3p*: *Rattus Norvegicus* microRNA-1 with 3p strand present in the reverse position; *rno-miR-30a-5p*: *Rattus Norvegicus* microRNA-30a with 5p strand present in the forward position; *rno-miR-208a-3p*: *Rattus Norvegicus* microRNA-208a with 3p strand present in the reverse position; *Cx26*: gap junction protein, beta 2 (Gjb2) or connexin 26 (Cx26); *RPL13*: ribosomal protein l13a.

## Data Availability

The data presented in this study are available on request from the corresponding author due to ethical reasons.
